# Corticosteroids in non-viral acute respiratory distress syndrome (ARDS): recommendations outpace evidence – a concise review

**DOI:** 10.1186/s41479-025-00183-x

**Published:** 2025-10-05

**Authors:** Thanh Luan Nguyen, Phuc Tuong Pham, Van Phieu Duong, Hoang Ngoc Thao Duong

**Affiliations:** 1 Faculty of Medicine, Nam Can Tho University, An Binh Ward, Can Tho, Viet Nam; 2 Critical Care Department, Nam Can Tho University Medical Center, Cai Rang Ward, Can Tho, Viet Nam

**Keywords:** Non-viral ARDS, Corticosteroid therapy, ARDS phenotypes

## Abstract

The 2024 global redefinition of acute respiratory distress syndrome (ARDS) broadens diagnostic criteria to include patients on non-invasive respiratory support. While this inclusivity enhances early recognition, it also introduces heterogeneity in disease severity, inflammatory burden, and treatment responsiveness–complicating the use of corticosteroids. Although recent guidelines recommend corticosteroids for moderate-to-severe ARDS, they fall short of specifying optimal timing, dosing, and patient selection. The DEXA-ARDS trial showed that high-dose dexamethasone reduced mortality in moderate-to-severe ARDS regardless of etiology, but its generalizability to less severe or non-intubated patients remains unclear. Conversely, in severe community-acquired pneumonia (CAP), hydrocortisone regimens used in CAPE-COD and APROCCHSS trials demonstrated mortality benefits, suggesting a particular therapeutic niche for corticosteroids in non-viral, infection-associated ARDS. A recent network meta-analysis suggests that low-dose methylprednisolone may reduce ARDS mortality, though phenotype-specific efficacy is not well defined. Despite these encouraging signals, key questions persist: Which corticosteroid? At what dose? For which ARDS phenotype? As evidence accumulates unevenly across ARDS subtypes, guideline-endorsed recommendations may inadvertently mask the nuances required for individualized therapy. Until precision approaches are better defined, clinicians must balance empirical benefit with thoughtful restraint in applying corticosteroids to non-viral ARDS. This concise review summarizes current evidence, key limitations, and pragmatic phenotype-informed strategies for corticosteroid use in non-viral ARDS.

## Introduction

The use of corticosteroids in acute respiratory distress syndrome (ARDS) has gained considerable traction, supported by randomized controlled trials (RCTs) [1–3] and recent guideline endorsements. The latest guidelines of American Thoracic Society (ATS) and Society of Critical Care Medicine (SCCM) recommend systemic corticosteroids for moderate-to-severe ARDS [4, 5]. However, ARDS is a syndrome with remarkable heterogeneity in its etiologies, with bacterial pneumonia being among the most common and clinically distinct subtypes [6, 7]. In this context, the application of corticosteroids becomes less a one-size-fits-all recommendation and more a question of phenotype-specific precision.

Recent advances suggest that ARDS is not a uniform entity but comprises distinct phenotypes and subphenotypes. Clinical phenotypes include direct versus indirect lung injury, focal versus non-focal radiographic patterns, and varying degrees of hypoxemia, reflecting different underlying pathophysiology [8]. Latent class analyses and biomarker-based studies consistently identify at least two biologic subphenotypes, “hyperinflammatory” and “hypoinflammatory,” which differ in cytokine profiles, frequency of shock and metabolic acidosis, ventilator-free days, mortality, and treatment responses [7, 9]. Recognizing this heterogeneity is crucial for interpreting corticosteroid trials and advancing more tailored therapeutic approaches. This framework provides a rationale to re-examine corticosteroid therapy in ARDS through the lens of phenotype-specific effects.

### ARDS from non-viral etiologies: a distinct inflammatory landscape

Non-viral ARDS encompasses a heterogeneous group of etiologies, including bacterial pneumonia, gastric aspiration, acute pancreatitis, extrapulmonary sepsis, and direct lung insults such as pulmonary contusion from trauma or transfusion-related acute lung injury [6, 7]. These diverse mechanisms induce ARDS through distinct inflammatory pathways. Bacterial pneumonia-induced ARDS, for example, involves a direct alveolar insult, intense neutrophilic infiltration, and a localized cytokine storm. This differs significantly from extrapulmonary causes, where systemic inflammation dominates [6, 7]. Understanding these immunopathological nuances is essential for tailoring corticosteroid therapy.

Evidence from meta-analyses and infection-focused trials lends credence to corticosteroid use in certain non-viral ARDS phenotypes. A systematic review and meta-analysis of 18 randomized controlled trials, including both COVID-19 and non-COVID-19 ARDS, demonstrated that corticosteroids were associated with a significant reduction in 28-day mortality (RR 0.82; 95% CI 0.72–0.95), with consistent benefits observed across etiologies and steroid regimens [10]. Notably, prolonged corticosteroid administration (>7 days) appeared more effective than shorter courses. These findings support a broader therapeutic role for corticosteroids in ARDS beyond viral contexts.

### Evidence gaps: between ARDS trials and infection trials

The CAPE-COD trial demonstrated that hydrocortisone significantly reduced mortality and improved clinical outcomes in patients with severe community-acquired pneumonia (CAP) [1]. Similarly, in a subgroup analysis of the APROCCHSS trial, patients with CAP-associated septic shock also benefited from hydrocortisone plus fludrocortisone [2]. Although these trials did not specifically enroll patients based on ARDS criteria, a substantial proportion of patients with severe CAP indeed fulfilled the Berlin [11] or newer ARDS definitions [12] during their clinical course.

In the CAPE-COD trial, 44% of patients assigned to hydrocortisone were enrolled while receiving positive pressure ventilation with a PaO_2_/FiO_2_ of 172, and 42% were on high-flow nasal oxygen with a PaO_2_/FiO_2_ of 130 [1]. All had pulmonary infiltrates, though the proportion with bilateral involvement was not reported. Thus, a large proportion likely met ARDS criteria under both Berlin and global definitions. In the APROCCHSS trial, 96% of patients were invasively ventilated at inclusion, with PaO_2_/FiO_2_ values between 175 and 200 [2]. While all had pulmonary infiltrates, the extent of bilateral involvement was not specified; however, most patients likely satisfied Berlin criteria. Importantly, no benefit of hydrocortisone was observed in patients with septic shock from non-pneumonia causes, underscoring the specificity of the effect in pneumonia-associated ARDS [2].

Moreover, in the DEXA-ARDS trial, dexamethasone significantly reduced mortality and increased ventilator-free days among patients with moderate-to-severe ARDS [3]. Pneumonia was the leading etiology, reported in 52% of patients in the dexamethasone group and 54% in the placebo group, while other causes such as aspiration, trauma, or extrapulmonary sepsis accounted for smaller proportions. Although the trial clearly established a benefit of dexamethasone in ARDS as a whole, outcomes were not stratified by etiology, leaving uncertainty regarding their efficacy in less common non-viral, non-pneumonia phenotypes.

These findings indicate that corticosteroid therapy in pneumonia-associated ARDS is supported by substantial indirect evidence. In contrast, patients with non-viral, non-pneumonia ARDS, such as those due to aspiration, pancreatitis, trauma, or transfusion, were either underrepresented or not analyzed as distinct subgroups in existing trials. Thus, recommendations are well supported for pneumonia-related ARDS but may overgeneralize when applied to other non-viral phenotypes. In practice, it is unlikely that future randomized trials will isolate “pure” non-viral, non-pneumonia ARDS populations, given both their relative rarity and the ethical challenges of withholding steroids in pneumonia. Therefore, the true evidence gap lies less in proving benefit for pneumonia-ARDS, which is reasonably established, and more in clarifying optimal regimens, timing, and applicability across less common non-viral phenotypes.

Given these gaps, a pragmatic timeline of corticosteroid use in non-viral ARDS phenotypes may help synthesize trial evidence into clinical practice (Table 1).

**Table 1:** Practical timeline for corticosteroid use in non-viral ARDS

Timing/clinical context	Typical regimen	Rationale/notes
Early severe CAP-associated ARDS or septic shock	Hydrocortisone 200 mg/day continuous infusion for 4–7 days, then taper; ± fludrocortisone in septic shock (CAPE-COD [1]; APROCCHSS subgroup [2]).	Anti-inflammatory and mineralocorticoid support; ↓ mortality, ↓ vasopressor use, ↓ intubation rates.
Early ARDS (≤ 72 h) with moderate-to-severe hypoxemia	Dexamethasone 20 mg daily × 5, then 10 mg daily × 5 (DEXA-ARDS [3])	Early anti-inflammatory effect; ↓ mortality; ↑ ventilator-free days.
Persistent inflammation or fibroproliferation (day 7–14)	Methylprednisolone 1–2 mg/kg/day × 7–14 days, then slow taper (meta-analyses [13])	Targets persistent inflammation or fibroproliferative ARDS; evidence modest but plausible.

Approximate glucocorticoid equivalence: hydrocortisone 20 mg ≈ methylprednisolone 4 mg ≈ dexamethasone 0.75 mg [14]

*ARDS* Acute respiratory distress syndrome, *CAP* Community-acquired pneumonia

### Tailoring corticosteroid regimens to ARDS etiologies

The optimal corticosteroid regimen may vary by ARDS phenotypes (Table 1). The DEXA-ARDS trial employed a relatively high dose of dexamethasone as 20 mg intravenous daily for 5 days, followed by 10 mg daily for 5 days, and demonstrated a significant reduction in 60-day mortality and an increase in ventilator-free days among patients with moderate-to-severe ARDS, regardless of underlying etiology [3]. These findings support the potential benefit of early, high-dose dexamethasone across etiologically diverse ARDS populations. The CAPE‑COD trial administered hydrocortisone 200 mg/day via continuous infusion started within 24 h of meeting severe CAP criteria, continued for 4–7 days, followed by tapering to 100 mg/day for 2–3 days and then 50 mg/day before discontinuation. This regimen significantly reduced 28-day mortality, vasopressor use, and intubation rate in severe CAP patients, many of whom likely had ARDS during their hospital course [1]. Additionally, in an a priori subgroup analysis of the APROCCHSS trial, the combination of hydrocortisone and fludrocortisone in patients with CAP‑related septic shock was associated with a 6% absolute risk reduction in 90‑day mortality, with a significant interaction compared to non‑CAP septic shock [2]. These regimens reflect a lower cumulative hydrocortisone and integrate both anti-inflammatory and mineralocorticoid effects, which may be particularly helpful in patients with hemodynamic instability.

Network meta-analyses have suggested that methylprednisolone, dosed at 1–2 mg/kg/day for 7 to 14 days, followed by a gradual taper over 6–10 days, may offer superior outcomes in general ARDS [13]. This approach is consistent with earlier RCTs and expert consensus, particularly for patients with persistent inflammation or fibrotic progression. However, these data are limited by small sample sizes and lack of etiologic stratification.

In bacterial ARDS or ARDS with septic shock, the mineralocorticoid activity of hydrocortisone may aid in vasopressor sparing and fluid regulation [2, 4, 14]. In contrast, dexamethasone’s long half-life and potent glucocorticoid effect may be advantageous in cases of severe hypoxemia without cardiovascular instability [3, 14]. Methylprednisolone, with its intermediate potency and pulmonary tissue penetration, may be better suited to patients with evolving fibroproliferative ARDS [13, 15]. These theoretical considerations, while biologically plausible, still require head-to-head clinical validation.

Timing remains critical. Both the latest ATS and SCCM guidelines recommend initiating corticosteroids within 72 h of ARDS onset [4, 5], particularly in moderate-to-severe disease, to maximize anti-inflammatory benefit and minimize progression. These guidelines largely derive from trials enrolling intubated patients under the Berlin definition, where ARDS onset is readily identifiable. However, with the expanded 2024 ARDS criteria, including non-intubated patients on high-flow oxygen, pinpointing the onset is more ambiguous. This uncertainty is amplified in cases with gradual inflammatory progression, such as sepsis or extrapulmonary injury. Accordingly, both guidelines underscore the importance of clinical judgment and individualized timing in corticosteroid initiation.

Furthermore, biomarkers to guide corticosteroid therapy in ARDS remain an area of growing interest. ARDS encompasses a spectrum of inflammatory responses, and differentiating hyperinflammatory from hypoinflammatory subphenotypes may hold the key to precision treatment [8]. In particular, the potential to use these markers not only for prognostication but also to determine the optimal timing, dosage, and candidacy for corticosteroids is being actively explored. While most evidence stems from viral or septic cohorts, the concept of inflammation-guided therapy is equally relevant in non-viral ARDS, where disease onset may be insidious and clinical phenotyping remains difficult. Future trials incorporating biomarker-based stratification may help bridge the gap between broad guideline recommendations and individualized steroid strategies.

### Toward a phenotype-guided strategy under the 2024 global definition

The 2024 global redefinition of ARDS now includes patients on high-flow nasal cannula or non-invasive ventilation with bilateral opacities and PaO_2_/FiO_2_ ≤ 300 mmHg [12]. While this inclusivity enhances early diagnosis, it also introduces heterogeneity in severity, inflammatory state, and therapeutic responsiveness, which complicates corticosteroid application.

Historically, most ARDS trials evaluated intubated patients, limiting direct evidence in non-invasively supported populations. However, in severe CAP-associated ARDS, the CAPE-COD trial enrolled nearly half of patients while on high-flow nasal oxygen, many of whom likely met the global ARDS definition, and demonstrated a mortality benefit from hydrocortisone [1]. This suggests that, for pneumonia-related ARDS, hydrocortisone efficacy may extend to patients managed with non-invasive respiratory support or mild intubated ARDS. In contrast, evidence remains limited for other non-viral ARDS phenotypes, particularly in non-intubated patients, leaving uncertainty about the generalizability of corticosteroid benefits beyond pneumonia.

Importantly, in groups where corticosteroid benefit is unproven, potential harm also appears limited. Risks vary with dose, duration, and host vulnerability, but contemporary evidence indicates overall safety when corticosteroids are used in appropriately selected patients [16].

Taken together, precision medicine in ARDS remains aspirational but necessary. Non-viral ARDS is a heterogeneous entity, and corticosteroid therapy must reflect this diversity. Current pragmatic strategies derive largely from indirect evidence and clinical reasoning (Table 2). In severe CAP-associated ARDS, hydrocortisone shows consistent mortality benefit, including in septic shock (CAPE-COD, APROCCHSS subgroup [1, 2]). Dexamethasone, validated in DEXA-ARDS, may be favored in non-septic patients with severe hypoxemia, though extrapolation beyond invasively ventilated cohorts should be cautious [3]. For other non-pneumonia phenotypes, evidence is sparse, and a cautious individualized approach remains essential. This phenotype-oriented framework, though imperfect, aligns therapy with both pathobiology and the best available evidence.

**Table 2:** Suggested pragmatic corticosteroid strategies in non-viral ARDS according to phenotype and severity

**Non-viral ARDS phenotypes**	**ARDS severity**			
	Non-invasive support	Intubated ARDS		
		Mild	Moderate	Severe
Severe CAP	Hydrocortisone (CAPE-COD [1])			
Severe CAP with septic shock	Hydrocortisone (CAPE-COD [1])		Hydrocortisone (APROCCHSS subgroup [2])	
Non-septic	Uncertain		Dexamethasone (DEXA-ARDS [3])	

*ARDS* Acute respiratory distress syndrome, *CAP* Community-acquired pneumonia

## Conclusion

Corticosteroid therapy in non-viral ARDS stands at a complex crossroad of evidence, guidelines, and pathophysiology. While trials in general ARDS and severe infections support their use, these data must be critically interpreted when applied to diverse non-viral phenotypes. Until future trials close these gaps, clinicians must anchor their decisions in a synthesis of available data, individual patient factors, and evolving ARDS definitions.

## Data Availability

No datasets were generated or analysed during the current study.

## Abbreviations

ARDS: Acute Respiratory Distress Syndrome
ATS: American Thoracic Society
CAP: Community-Acquired Pneumonia
COVID-19: Coronavirus Disease 2019
RCT: Randomized Controlled Trial
RR: Relative Risk
SCCM: Society of Critical Care Medicine
